# *In vivo* evidence for glycyl radical insertion into a catalytically inactive variant of pyruvate formate‐lyase

**DOI:** 10.1002/1873-3468.70075

**Published:** 2025-05-19

**Authors:** Michelle Kammel, A. F. Volker Wagner, R. Gary Sawers

**Affiliations:** ^1^ Institute for Biology/Microbiology Martin Luther University Halle‐Wittenberg Germany

**Keywords:** anaerobic metabolism, glycyl radical enzymes, half‐site reactivity, oxygenolytic cleavage, pyruvate formate‐lyase, radical transfer

## Abstract

The dimeric glycyl radical enzyme pyruvate formate‐lyase (PflB; formate acetyltransferase 1) cleaves pyruvate with hypothetical half‐site reactivity to formate and acetyl‐CoA. The radical introduced onto residue G734 of PflB is transiently transferred to C419 of an adjacent cysteine pair (C418/C419) during catalysis, but it is unclear whether glycyl radical formation is dependent on C419 *in vivo*. We show here that a deficiency in formate production of an *Escherichia coli* strain synthesizing a PflB_G734A_ variant, but lacking the autonomous glycyl radical cofactor, GrcA, could be restored by reintroducing plasmid‐encoded native PflB, but not by a PflB_C418A/C419A_ variant, indicating that PflB_C418A/C419A_ cannot replace GrcA. Oxygen‐dependent polypeptide cleavage of PflB_C418A/C419A_ indicated stable glycyl radical incorporation; however, these data did not support half‐site reactivity. These *in vivo* findings demonstrate that glycyl radical formation is independent of subsequent radical transfer from G734 to C419, which occurs intramolecularly.

**Impact statement**

Active, dimeric pyruvate formate‐lyase has a stable radical on a glycine residue, which transiently abstracts a H‐atom from a cysteine, generating a catalytic thiyl radical. Glycyl radical generation is independent of glycine‐to‐cysteine radical‐transfer *in vivo*. Radical‐transfer is intramolecular and the enzyme does not appear to exhibit half‐site reactivity.

## Graphical abstract

Dimeric pyruvate formate‐lyase cleaves pyruvate using a radical‐based mechanism. G734 serves as a radical storage location, and the radical is transferred to the catalytic C419 residue. Mutation of the C418‐C419 pair causes loss of enzyme activity, but does not impede radical introduction onto G734. Therefore, *cis*‐ but not *trans*‐radical transfer occurs *in vivo* and radical storage is independent of catalysis.

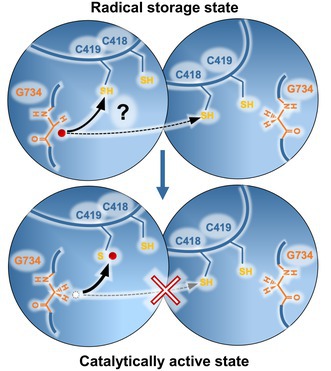

## Abbreviations

**CoA**, coenzyme A

**FHL**, formate hydrogenlyase

**GrcA**, glycyl radical cofactor A

**GRE**, glycyl radical enzymes

**PflA**, pyruvate formate‐lyase‐activating enzyme

**PflB**, pyruvate formate‐lyase

A signature reaction of fermentative metabolism in enterobacteria is the coenzyme A (CoA)‐dependent homolytic cleavage of pyruvate to acetyl‐CoA and formate, catalyzed by the glycyl radical enzyme (GRE), pyruvate formate‐lyase (PflB) [1, 2, 3]. The radical‐based cleavage of pyruvate obviates the generation of reduced pyridine nucleotide [4] and instead reducing equivalents remain associated with formate. Direct excretion of formate together with a proton from the cell by the formate channel, FocA [5], or its disproportionation to H_2_ and CO_2_, catalyzed by the formate hydrogenlyase (FHL) complex [6], allows a simple means of jettisoning these reducing equivalents.

PflB is a homodimer and, when synthesized, is catalytically inactive. The radical is introduced into a *C*‐terminal domain on the polypeptide by pyruvate formate‐lyase‐activating enzyme (PflA), which belongs to the superfamily of radical *S*‐adenosylmethionine enzymes [7, 8, 9]. PflA generates a 5'‐deoxyadenosyl radical, which stereospecifically abstracts the pro*‐S* H‐atom from a highly conserved glycine residue at position 734 (G734) on the polypeptide chain of PflB [3, 10, 11]. The G734‐radical is kinetically stable, and G734 thus serves as the location for radical storage [3]. The catalytic cycle requires H‐atom abstraction by the radical on G734 from the adjacent active‐site cysteine residue C419, where the transient generation of a thiyl radical allows initiation of pyruvate cleavage to take place [12, 13].

As is hypothesized to be the case for other GREs [14, 15, 16], PflB appears to show half‐site reactivity *in vitro*, whereby only one protomer per homodimer carries the glycyl radical [12, 13]. Half‐site reactivity is common in enzymology, particularly when the enzyme is functional as a dimer, but the rationale behind half‐site reactivity is still poorly understood [17]. It is likely that it allows better regulatory control over catalytic activity. Nevertheless, how this form of reactivity is imposed is also unclear. While recent structural studies with aerobic ribonucleotide reductase [18] indicate asymmetric binding of the two subunits of the homodimer, supporting an early hypothesis for half‐site reactivity [19], it is also conceivable that for some enzymes a form of allosteric regulation may be involved in preventing radical insertion or catalytic activity of the second subunit [17].

In the case of PflB, half‐site reactivity in terms of *in vitro* radical insertion was confirmed by electron spin resonance quantification of radical content [12], and further supported by enzyme purification studies, which revealed polypeptide scission of the radical‐bearing monomer [3]. The two polypeptide species purified exhibited near‐stoichiometry but had molecular masses that differed by approximately 2–3 kDa [3]. The extreme oxygen sensitivity of the glycyl radical results in the chemical cleavage of the polypeptide backbone between residues S733 and G734 on PflB during enzyme isolation, which has proved to be a useful phenotype facilitating the analysis of radical insertion into PflB [3, 20]. This oxygen sensitivity of GREs is a general problem for facultative anaerobic bacteria such as *E. coli*, which are frequently exposed to O_2_. Remarkably, these bacteria have evolved a small protein, termed GrcA, that functions as an autonomous glycyl radical cofactor [21, 22, 23]. GrcA has a conserved glycyl radical domain similar to that of PflB and functions together with PflA to interact with and rescue the activity of oxygen‐damaged PflB [21, 22]. The radical introduced on residue G102 of GrcA (based on *E. coli* numbering) subsequently abstracts the H‐atom intermolecularly from C419 of PflB to restore catalytic activity [21, 22, 23, 24].

**Fig. 1:**
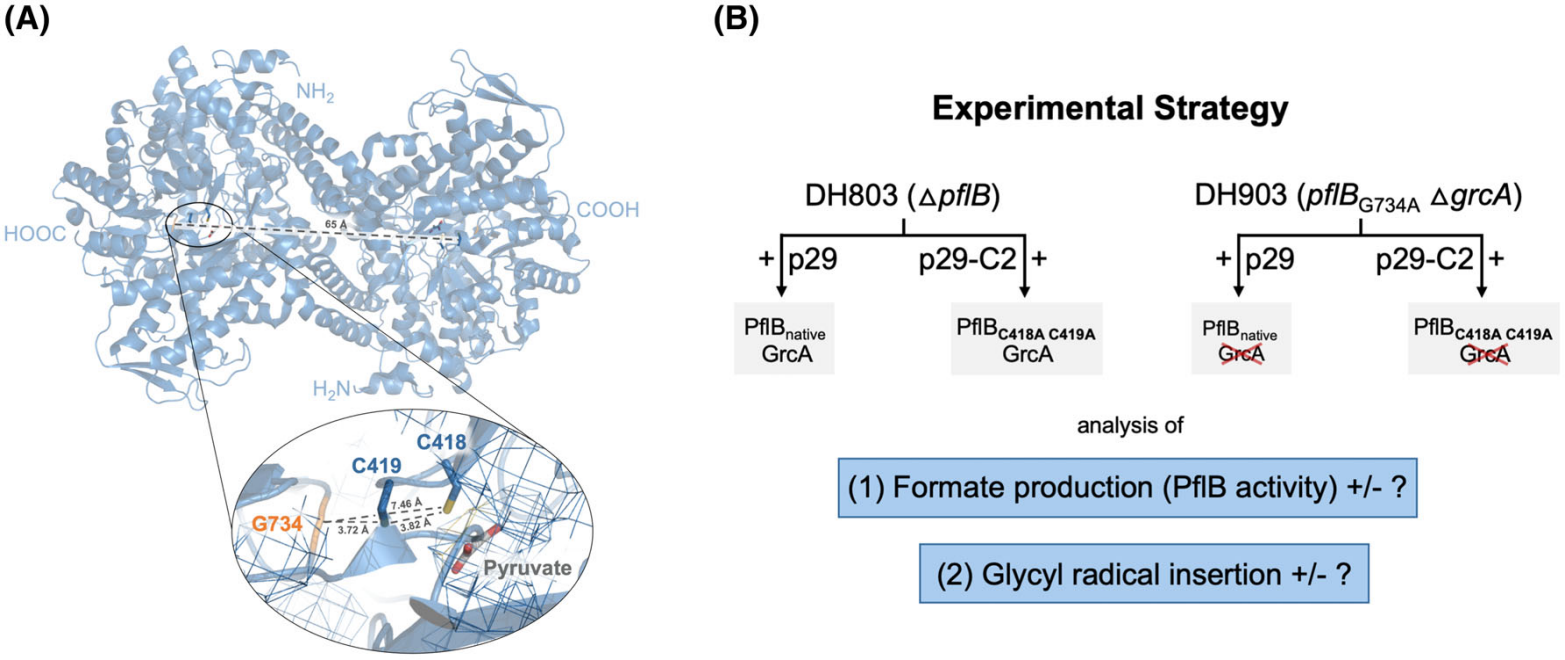
Structural representation of PflB and experimental strategy to assess radical transfer within dimeric PflB. Panel (A) shows the structure of dimeric PflB (Pdb: 1H18 [25]). Side chains of the amino acid residues involved in radical storage (G734), catalysis (C418, C419) and the binding of PflB's substrate pyruvate are highlighted. The distances between key residues are indicated. The inset zooms in to show the proximity between G734 and C418–C419, with electron density shown in mesh representation. Structures were rendered with PyMOL Molecular Graphics System, version 2.5, Schrodinger, LLC. Panel (B) gives a schematic overview of the experimental strategy used to investigate radical insertion and activity of PflB *in vivo* and its amino acid residue variants (see first section of Results and Discussion for further details). Strain DH903 lacks the autonomous glycyl radical cofactor, GrcA, which is signified by the red cross.

The fact that GrcA can abstract a hydrogen atom from C419 in PflB, which lacks an intact *C*‐terminal domain [21, 22], raises the question as to whether the radical on G734 in PflB is only transferred to the proximal catalytic cysteine pair, which lies within 3 to 4 Å from G734 (Fig. 1A), or whether indeed the radical can be transferred to the C419 cysteine residue in the other subunit, which lies at a distance of > 65 Å, based on structural analyses [26]. Despite long‐range (32 Å) radical transfer occurring in aerobic ribonucleotide reductase [27], such a distance within the PflB homodimer for radical transfer would be unprecedented. A second question that arises is whether a stable radical on G734 can be introduced if the radical cannot be subsequently transferred to C419 residues within the protein. Evidence for the *in vitro* generation of such a radical on G734 of a PflB_C419S_ variant has recently been provided [28], but it is unclear whether this also occurs *in vivo*. By using biochemical, genetic, and physiological analyses, we address these two questions for *E. coli* PflB. Our *in vivo* experimental findings support intramolecular H‐atom abstraction by the radical on G734 of PflB, and blocking radical transfer to C419 by mutation does not impede radicalization of G734. Moreover, our studies present evidence that does not support half‐site reactivity of PflB.

## Materials and methods

### Bacterial strains, plasmids, and general growth conditions

The strains used in this study are isogenic derivatives of MC4100 (*F‐ araD139 Δ(argF‐lac)U169 ptsF25 deoC1 relA1 flbB5301 rspL150* ^−^) [29] and included DH4100 (MC4100 carrying a chromosomal λ*fdhF*_*P*_ *::lacZ* fusion; Km^r^) [30], DH801 (like DH4100, but with the glycine codon 735 changed to an alanine codon, GCA in *pflB*) [24], DH903 (like DH801, but Δ*grcA*) [24] and DH803 (like DH4100, but Δ*pflB*) was generated by transfection of strain MC803 [24] with a lambda phage carrying *fdhF*_*P*_::*lacZ*. Note that the *E. coli pflB* gene has 760 codons (excluding the stop codon), but the PflB protein lacks the formyl‐methionine residue and thus has only 759 amino acid residues [31]. Historically [3], the catalytically active glycyl residue is therefore numbered 734 and not 735.

The plasmids used included p29 (*focA* ^+^ *pflB* ^+^ *pflA* ^+^, Cm^r^) [32] and its derivative p29‐C2 (*pflB* codons 419 and 420, both TGC and coding for cysteine, exchanged for GCC coding for alanine) [24]. Expression of the *focA‐pflB* operon and the *pflA* gene on the plasmids was under the control of their respective native promoters [32].

Cells were grown exactly as described [33] using anaerobic cultivation in M9‐minimal medium [34] with 0.8% (w/v) glucose as a carbon source and in standing‐liquid cultures at 37 °C.

When required, antibiotics were used at the following final concentrations: 100 μg/mL for kanamycin and 25 μg/mL for chloramphenicol.

### Polyacrylamide gel electrophoresis (PAGE) and immunoblotting

Plasmid‐based overproduction of PflB was investigated in *pflB* and *grcA* mutant strains that were grown anaerobically as described above. Harvest of cells and preparation of crude, cell‐free extracts were carried out exactly as described [24]. Samples ranging from 10 to 50 μg of protein were separated by denaturing SDS/polyacrylamide gel electrophoresis (SDS/PAGE) using 8% (w/v) polyacrylamide PRiME™ SERVA*Gels* (SERVA, Heidelberg Germany). To allow optimal separation of polypeptides in the range between 55 and 120 kDa, the gel was run until proteins with a molecular mass smaller than 55 kDa had migrated through the gel. Silver staining of the gel followed the instructions provided in the Pierce Silver‐staining kit (Thermo Fisher Scientific). Immunodetection analysis using anti‐PflB antiserum, which was generated using the full‐length protein and used at a dilution of 1:3000, proved PflB's identity and its glycyl radical‐bearing state [24]. Chemoluminescence was detected using the Amersham™ Imager 600 (GE Healthcare, Freiburg, Germany) and the intensities of signals were investigated using its analysis software package.

### Analysis of intracellular formate concentrations

All strains used in this study carried a genomically integrated *fdhF*_*P*_::*lacZ* fusion. This *lacZ*‐based reporter system was used to determine changes in intracellular formate levels in response to the introduced mutations or plasmids [33, 35]. Cells were cultivated in 15 mL Hungate tubes in M9‐minimal medium containing 0.8% (w/v) glucose at 37 °C. Samples for the analysis of intracellular formate levels were taken when cells reached the mid‐ to late‐exponential phase (OD_600nm_ ~ 0.7–0.85) of growth, and the *ß*‐galactosidase enzyme activity for these whole cells was determined as described [33].

The assays were carried out in triplicate with minimally three biological replicates and data, shown in Miller units [36], are presented with the standard deviation of the mean.

### Analysis of extracellular formate and lactate levels

To correlate extracellular formate levels with changes in the respective intracellular level, the same cultures as those used to determine *ß*‐galactosidase enzyme activity were used to measure the concentration of formate and lactate in the culture medium [33]. The quantification of fermentation products by high‐performance liquid chromatography (HPLC) was done in triplicate with minimally three biological replicates. Concentrations are presented with reference to the optical density (OD_600nm_) of the culture.

### Analysis of H_2_ production via gas chromatography

Quantification of H_2_ gas produced by the formate‐dependent FHL complex was done exactly as described [25] after strains had been cultivated anaerobically in 15 mL Hungate tubes (with 10 mL headspace) in M9‐glucose minimal medium at 37 °C for 24 h. The accumulated amount of H_2_ formed was determined by gas chromatography. Each experiment was done in triplicate, with a minimum of three biological replicates. The amount of H_2_ was calculated, and the data were presented with reference to the optical density (OD_600nm_) of the culture.

### Computational analysis

Visualization of the crystal structure of the *E. coli* PflB dimer and its catalytic residues (pdb: 1H18 [37]) was done using PyMOL (The PyMOL Molecular Graphics System, version 2.5, Schrodinger, LLC). The structure is shown in cartoon and mesh representations with relevant residue side‐chains and the substrate pyruvate represented in stick form. The PyMOL algorithm was also applied for the determination of distances between amino acid residues.

## Results and Discussion

### Experimental strategy to test for radical insertion onto glycine residue 734 and catalytic activity of PflB *in vivo*

The proximity of G734, the radical storage location on PflB [37], and the catalytic C418 and C419 residues (Fig. 1A) indicates that direct, intramolecular radical transfer from G734 to C419 (G734 to C419_SH_ = 3.72 Å) is feasible. However, it is theoretically conceivable that if transfer of the radical to the proximal C419 residue [28, 38] is impeded, for example through mutation, then inter‐subunit radical transfer to the C419 residue in the associated protomer might occur, although highly improbable due to distance constraints (distance between G734 on chain A and C419 on chain B = 65 Å) (Fig. 1A). It is important to test this possibility because an intermolecular radical transfer occurs between G102 on GrcA and C419 in PflB, albeit a transfer that presumably occurs over a short molecular distance [22, 24]. An experimental strategy was designed to test whether only intramolecular (i.e., intra‐subunit) radical transfer occurs within PflB, or if inter‐subunit radical transfer is possible *in vivo*, as shown in Fig. 1B. These experiments involved using two *E. coli* strains: strain DH803 lacks a genomic copy of the *pflB* gene, and therefore cannot synthesize PflB, but retains a wild‐type *grcA* gene [24]; strain DH903 lacks *grcA*, has a genomic copy of *pflB*, which carries a G734A mutation preventing generation of a radical at this position and is therefore catalytically inactive in production of formate and acetyl‐CoA [10, 24]. Strains DH803 and DH903 were then transformed with either one of two plasmids: p29, which synthesizes native FocA, PflB, and PflA [32]; or p29‐C2, which encodes a PflB enzyme with the adjacent catalytic cysteine residues, C418 and C419, converted to alanine residues [24]. Thus, this p29‐C2 plasmid encodes native FocA and PflA, but a PflB_C418A/C419A_ variant that cannot cleave pyruvate to acetyl‐CoA and formate [24].

**Fig. 2:**
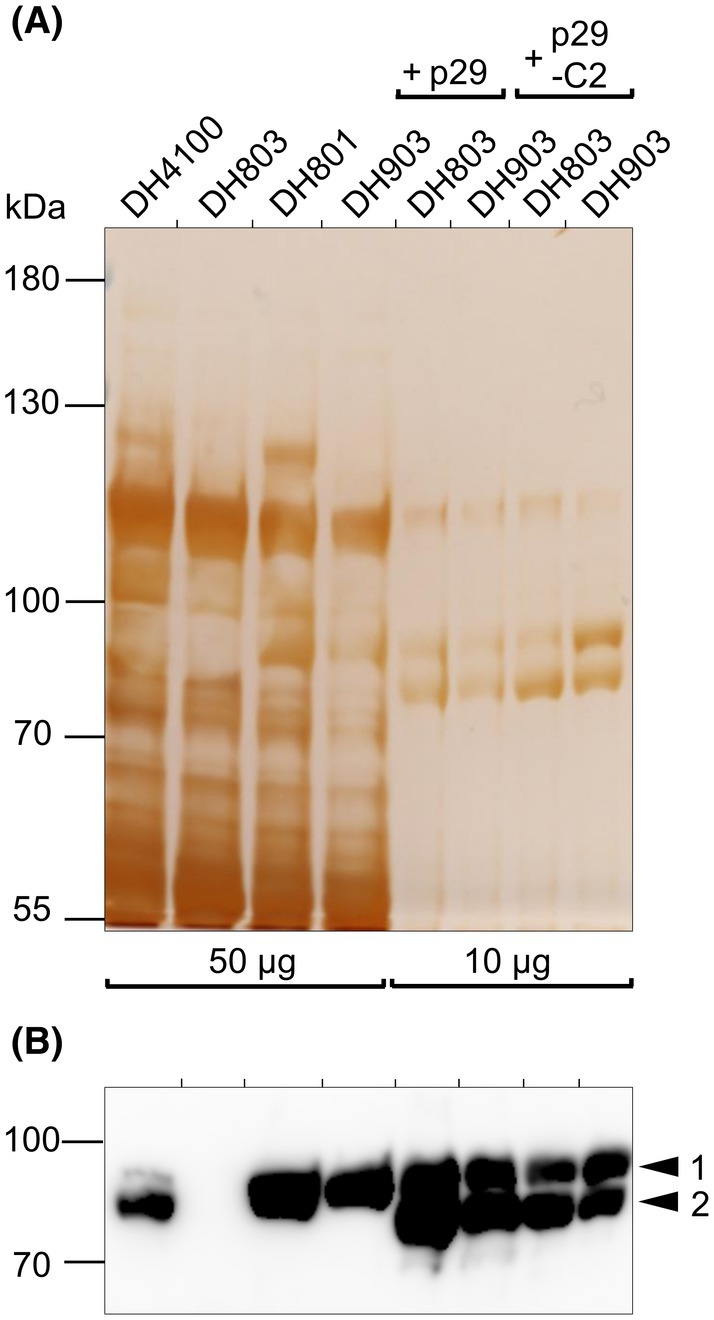
Glycyl radical insertion into a plasmid‐encoded PflB_C418A/C419A_ variant. (A) Polypeptides in crude extracts derived from the indicated strains (50 μg of protein for DH4100, DH803, DH801, and DH903; 10 μg of protein for DH803 or DH903 cells transformed with p29 and p29‐C2) were separated in denaturing SDS/PAGE (8% w/v polyacrylamide) and the gel was subsequently silver‐stained. (B) A portion of an immunoblot of a similar gel run in parallel to the one in (A) is shown after the blot was treated with anti‐PflB antiserum. The arrows on the right of the blot indicate full‐length, native PflB (1) and the oxygenolytically cleaved PflB polypeptide (2) resulting from exposure of glycyl radical‐bearing PflB to O_2_ during cell breakage. In both panels, the migration positions of molecular mass markers (PageRuler prestained protein ladder; Thermo Fisher Scientific) are indicated in kDa on the left. The experiments shown in both panels were each repeated once, with the same results.

**Fig. 3:**
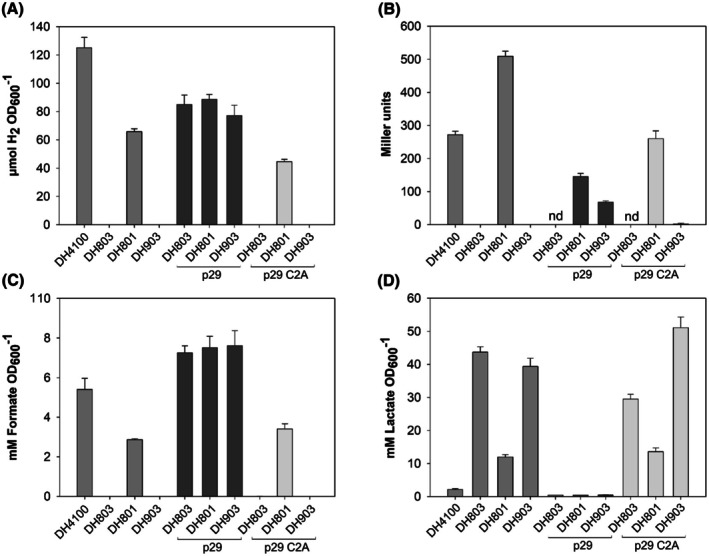
In the absence of GrcA, PflB activity can only be restored by radical transfer from G734 to C419 *in cis*. (A) PflB activity in the indicated strains was investigated physiologically by determining extracellular formate levels and, (B), by monitoring changes in intracellular formate levels using a *β*‐galactosidase‐based reporter system [35], presented in Miller units [36]; nd, not determined. (C) Determination of the extracellular lactate concentration after anaerobic growth to the mid‐ to late‐exponential phase was performed for each of the indicated strains and gives an indirect indication of PflB activity; increased lactate production is indirectly proportional to PflB activity. (D) Shown is the amount of H_2_ that accumulated in the gas phase after 24 h of fermentative growth of the indicated strains. All indicated strains carry a chromosomally integrated formate‐dependent *fdhF*_*P*_::*lacZ* fusion to allow direct monitoring of changes in intracellular formate levels, measured as *β*‐galactosidase enzyme activity [36]. Strains included: the parental strain, DH4100; DH803, a *pflB* deletion mutant; DH801, encoding PflB_G734A_; DH903, encoding PflB_G734A_, but also carrying a deletion in *grcA*. Where indicated, strains were transformed with either plasmid p29, encoding native FocA, PflB, and PflA, or plasmid p29‐C2, encoding native FocA and PflA, but a PflB_C418A/C419A_ variant. All experiments were performed with three biological replicates, each with three technical replicates. Data are shown as the standard deviation of the mean.

Along with these transformed strains, the *E. coli* control strains used included DH803 and DH903 without a plasmid, the isogenic parental strain DH4100, and strain DH801 that synthesizes the PflB_G734A_ variant, but which has a wild‐type genomic copy of the *grcA* gene [24]. The phenotypic characteristics of each strain, with respect to the PflB and GrcA proteins, and to formate production, are summarized in Table 1. After anaerobic growth of the strains to late‐exponential phase, immunoblot analysis (Fig. 2) was used to demonstrate whether PflB was synthesized and whether the cleaved species of the glycyl radical‐bearing PflB polypeptide was generated [3, 20]. Extracellular as well as intracellular formate levels, together with H_2_ gas accumulation [25], were determined as indicators of the ability of the PflB derivative to cleave pyruvate or not (Fig. 3).

**Table 1:** Relevant phenotypic characteristics of the strains.

Strain^a^	Proteins present	Ability to produce formate^b^
DH4100	PflB, GrcA	**+++**
DH801	PflB_G734A_, GrcA	**++**
DH803	No PflB, GrcA	**−**
DH903	PflB_G734A_, no GrcA	**−**

^a^ All strains synthesize genomically encoded PflA.

^b^ +++, high; ++, intermediate; −, no.

### Incorporation of a glycyl radical into the PflB_C418A_ _/C419A_ variant

Empirical SDS/PAGE analysis showed that in crude extracts derived from the plasmid‐bearing strains, roughly five‐fold higher amounts of PflB were synthesized compared with the levels of PflB present in plasmid‐free strains (Fig. 2A). Immunoblotting with anti‐PflB antiserum (Fig. 2B) identified two polypeptides in the extract derived from the parental strain DH4100, with the slightly faster‐migrating polypeptide representing the oxygenolytically cleaved polypeptide that is 3 kDa smaller than full‐length PflB (Fig. 2B [3]). Notably, the stoichiometry of the full‐length versus oxygenolytically cleaved PflB fragment in extracts of DH4100 (parental strain, Table 1) was approximately 1:5 (based on densitometric scanning of the blot) in favor of the cleaved fragment (Fig. 2B). This observation argues against half‐sites reactivity of PflB *in vivo*. Analysis of an extract derived from strain DH803 clearly showed that it lacked PflB. Extracts derived from the untransformed strains DH801 (PflB_G734A_^+^) and DH903 (GrcA^−^; PflB_G734A_^+^) synthesized PflB_G734A_ that migrated as a single polypeptide species at approximately 85 kDa, representing the full‐length polypeptide. Strain DH801 does not generate an oxygenolytically cleaved PflB fragment because it cannot store the radical on A734 [3, 20].

Western blot analysis of cell‐free extracts derived from strains DH803 (PflB^−^) and DH903 (GrcA^−^; PflB_G734A_^+^) transformed with p29 or p29‐C2 all showed a double band (Fig. 2B); however, again, the slight accumulation of the cleaved relative to full‐length polypeptide further argues against half‐site reactivity of PflB. The extract derived from DH903 transformed with plasmid p29‐C2, which synthesizes PflB_G734A_ from its genome, plus PflB_C418A/C419A_ from p29‐C2, revealed a roughly stoichiometric distribution of the two polypeptides (Fig. 2B). As this double‐band signature is characteristic of the presence of a glycyl radical immediately prior to cell breakage, it can be concluded from this experiment that PflB_C418A/C419A_ carried a glycyl radical and this was the origin of the cleavage. This conclusion can be drawn because strain DH903 synthesizes the PflB_G734A_ variant that cannot be activated by PflA (Fig. 2B, lane 4). Consequently, these results show that the G734 residue on the PflB_C418A/C419A_ variant carries a radical *in vivo* (Fig. 2B, lane 8 – DH903/p29‐C2), which corroborates analogous *in vitro* studies [28]. Moreover, although the stoichiometry of the two bands identified for the PflB_C418A/C419A_ variant supports half‐site reactivity *in vivo*, the findings for the native PflB protein in DH4100 (Fig. 2B, lane 1) do not.

### The glycyl radical‐bearing PflB_C418A_ _/C419A_ variant is catalytically inactive *in vivo*

Analysis of extracellular formate concentrations in the culture medium showed that the parental strain DH4100 (PflB^+^) excreted 5.4 ± 0.6 mM formate per OD_600_^−1^ (Fig. 3A), while strains DH803 (PflB^−^) and DH903 (GrcA^−^; PflB_G734A_^+^) produced no formate, as expected [24] (Table 1). In contrast, DH801 (PflB_G734A_^+^) retained the ability to generate formate at approximately 50% of the level produced by the parental strain, DH4100 (Fig. 3A). These results were confirmed by determining intracellular formate production (Fig. 3B) through the *β*‐galactosidase enzyme activity that was generated from the formate‐responsive reporter (*fdhF*_*P*_::*lacZ* fusion) present on the chromosome of each strain [24, 30].

If pyruvate cannot be cleaved because PflB is inactive, it is reduced to lactate, which is excreted from the cell in high amounts [24, 39]. While strain DH4100 with an active PflB enzyme excreted the comparatively low amount of 2.1 ± 0.2 mM lactate into the culture medium, strains DH803 (PflB^−^) and DH903 (GrcA^−^; PflB_G734A_^+^) excreted approximately 20 times this level of lactate (Fig. 3C), confirming that pyruvate could not be cleaved to formate because PflB was either not synthesized or could not be activated by PflA. Lactate levels determined for strain DH801 (PflB_G734A_^+^) were roughly 4‐fold lower than for DH803 (PflB^−^), but were still 5.6‐fold higher than for the parental strain (Fig. 3C), indicating that while the catalytic activity of PflB_G734A_ was rescued by GrcA, it apparently did not attain the levels of the wild‐type enzyme.

A further final control was the measurement of H_2_ gas that accumulates during fermentative growth of *E. coli* on glucose [2, 6]. Strain DH4100 accumulated 125 ± 7 μM H_2_, while strains DH803 and DH903 accumulated no detectable H_2_ (Fig. 3D). Strain DH801 produced approximately half the amount of H_2_ compared with the wild‐type strain, again consistent with an apparent lower pyruvate formate‐lyase activity of the strain.

As expected, introduction of plasmid p29 (*focA* ^+^ *pflB* ^+^ *pflA* ^+^) into strains DH803 and DH903 restored the ability of both strains to produce formate, which was present at high extracellular concentrations (Fig. 3A). This correlated with high levels of accumulated H_2_ gas through the activity of the FHL complex (Fig. 3D). Notably, the amount of H_2_ formed by the p29‐bearing strains was approximately 30%–40% lower than that of the parental strain, suggesting that coordinated overproduction of FocA, PflB, and PflA is not suitable to allow greater H_2_ production, presumably because intracellular formate is maintained at a lower level (Fig. 3B) through the formate/H^+^‐efflux activity of FocA [33].

When the three strains were transformed with plasmid p29‐C2, encoding FocA, PflA, and PflB_C418A/C419A_, and formate production was determined, only strain DH801/p29‐C2 excreted formate, and this was at a level slightly higher than that for DH801 without a plasmid (Fig. 3A). Neither strain DH803 nor strain DH903 transformed with plasmid p29‐C2 had the capability of generating H_2_ (Fig. 3D) and because pyruvate could not be cleaved, these strains instead reduced pyruvate to lactate (Fig. 3C). The lack of restoration of formate or H_2_ production by introducing p29‐C2 into strain DH903 strongly suggests that no inter‐subunit radical transfer from G734 to C419 was possible, even if, in the unlikely event, a heterodimer comprising one subunit of PflB_A734_ and the other subunit of PflB_A418/A419_ could form. These findings demonstrate that, *in vivo*, apparently only intramolecular radical transfer between radical‐bearing G734 and C419 within the PflB protein occurs during the catalytic cycle. Although our findings do not completely rule out that the C419‐thiyl radical on chain A undergoes reversible exchange with the thiolate of C419 on chain B in native PflB, we consider this possibility implausible due to the > 60 Å separation between the two thiolates. Moreover, although the PflB structure (pdb: 1H18) does show a potential radical transfer pathway comprising tyrosine residues between the two C419 residues, as observed for ribonucleotide reductase [18], the route is rather indirect and has distances of > 15 Å between some residues (Fig. S1), making electron transfer very inefficient.

## Conclusions

This *in vivo* study shows that the ability of PflA to abstract the hydrogen atom from G734 on PflB is not dependent on whether further transfer of the radical to the active‐site cysteine residue, C419, can occur, as also recently demonstrated during *in vitro* activation of the enzyme [28]. Moreover, the proximal intramolecular radical transfer between G734 and C419 is PflB strongly suggests that when GrcA rescues oxygen‐damaged PflB [21, 22, 24], delivery of the *S*‐adenosylmethionine‐dependent radical generated by PflA to PflB via GrcA requires PflA and GrcA to displace the damaged radical domain of PflB so that proximal intermolecular radical transfer directly from G102 on GrcA to C419 on PflB can occur, as proposed [21, 22, 23].

The hypothetical half‐site nature of glycyl radical insertion into PflB was not found to be supported by the immunological data presented in the current study, whereby non‐stoichiometric accumulation of the oxygenolytically cleaved polypeptide form was clearly observed for the native PflB enzyme. This finding substantiates earlier observations made for both PflB [20, 24, 40] and for benzylsuccinate synthase [16], where the radical‐dependent cleavage product was detected in higher abundance compared with the full‐length polypeptide. These data suggest that the apparent half‐site reactivity of PflB may result from incomplete activation or from radical quenching *in vivo*.

## Author contributions

M.K., A.F.V.W., and R.G.S. conceived the study. R.G.S. led the study and secured funding. All experimental work was performed by M.K. All authors provided intellectual input by analyzing and discussing the data. M.K., A.F.V.W., and R.G.S. wrote the manuscript. All authors discussed and read the manuscript and provided critical feedback before its submission.

## Conflicts of interest

The authors declare no conflicts of interest.

## Peer review

The peer review history for this article is available at https://www.webofscience.com/api/gateway/wos/peer‐review/10.1002/1873‐3468.70075.

## Supporting information


**Fig. S1.** Potential pathway within dimeric PflB to facilitate inter‐subunit radical transfer.

## Data Availability

Any additional data that support the findings of this study are available upon reasonable request from the corresponding author at: gary.sawers@mikrobiologie.uni-halle.de.
