# Supplementary material for: In vivo evidence for glycyl radical insertion into a catalytically inactive variant of pyruvate formate‐lyase
Source: FEBS Lett. 2025 May 19;599(15):2201–9. doi: 10.1002/1873-3468.70075 (PMC12338860; doi:10.1002/1873-3468.70075)
Supplement: Supplementary file 1 — Fig. S1. Potential pathway within dimeric PflB to facilitate inter‐subunit radical transfer. [file FEB2-599-2201-s001.pdf]

Supplementary Information

In vivo Evidence for Glycyl Radical Insertion into a Catalytically Inactive Variant of Pyruvate Formate-Lyase

Michelle Kammel[1], A. F. Volker Wagner[1], and R. Gary Sawers[1*]

[1]Institute for Biology/ Microbiology, Martin Luther University Halle-Wittenberg, Kurt-Mothes-Str. 3, 06120 Halle (Saale), Germany

[*]Address correspondence to: R. G. Sawers, Institute for Biology/Microbiology, Martin Luther University Halle-Wittenberg, Kurt-Mothes-Str. 3, 06120 Halle (Saale) Germany; phone +49 345 5526350; Fax. +49 345 5527010; Email gary.sawers@mikrobiologie.uni-halle.de

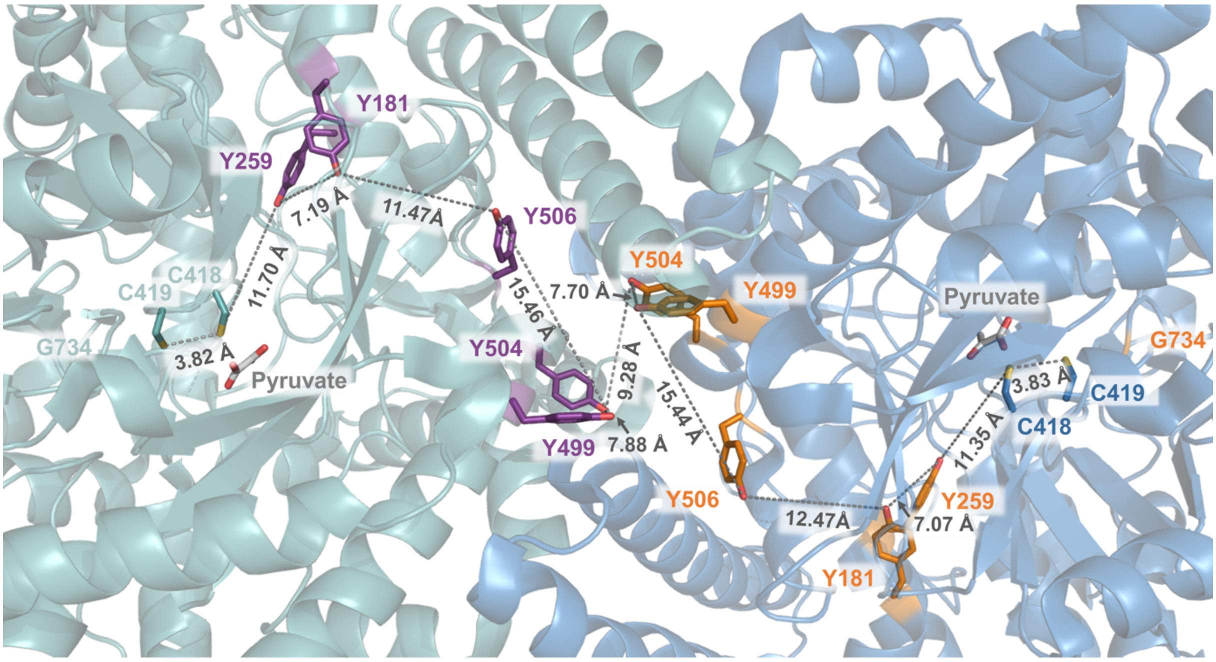

Figure S1. Potential pathway within dimeric PflB to facilitate inter-subunit radical transfer. Depicted is a portion of the structure of dimeric PflB (monomers are in teal and sky blue) showing both catalytic centres (Pdb: 1H18, [1]). Besides the side chains of C418, C419 and G734, various tyrosine residues within a broad radical transfer distance (< 16 Å) are highlighted in stick format. The distances between the residues were determined with the PyMOL algorithm and are indicated. The structure was rendered with PyMOL Molecular Graphics System, version 2.5, Schrodinger, LLC.

Reference

1. Becker A and Kabsch W (2002) X-ray structure of pyruvate formate-lyase in complex with pyruvate and CoA: How the enzyme uses the Cys-418 thiyl radical for pyruvate cleavage. *J Biol Chem* **277**, 40036–40042.
